# Local Oral Delivery Agents with Anti-Biofilm Properties for the Treatment of Periodontitis and Peri-Implantitis. A Narrative Review

**DOI:** 10.3390/molecules26185661

**Published:** 2021-09-17

**Authors:** Shorouk Elnagdy, Michail Raptopoulos, Ioannis Kormas, Alessandro Pedercini, Larry F. Wolff

**Affiliations:** 1 Division of Periodontology, Department of Developmental and Surgical Sciences, School of Dentistry, University of Minnesota, Minneapolis, MN 55455, USA; rapto003@umn.edu (M.R.); korma059@umn.edu (I.K.); pede0783@umn.edu (A.P.); wolff001@umn.edu (L.F.W.); 2 Department of Periodontics, College of Dentistry, Texas A&M University, Dallas, TX 75246, USA

**Keywords:** biofilm, oral cavity, periodontitis, peri-implantitis, anti-biofilm

## Abstract

Despite many discoveries over the past 20 years regarding the etiopathogenesis of periodontal and peri-implant diseases, as well as significant advances in our understanding of microbial biofilms, the incidence of these pathologies continues to rise. For this reason, it was clear that other strategies were needed to eliminate biofilms. In this review, the literature database was searched for studies on locally delivered synthetic agents that exhibit anti-biofilm properties and their potential use in the treatment of two important oral diseases: periodontitis and peri-implantitis.

## 1. Introduction

During the last two decades it has been shown that the biofilm comprises the predominant life-mode of most bacterial species [1]. Biofilms are complex structures where bacteria are usually densely packed in microcolonies and protected in a matrix of biopolymers [2]. In humans, bacteria are able to form biofilms at various body sites, and with the extensive use of medical devices in modern health care, this has provided even more new niches for bacterial biofilm formation and chronic infections.

Biofilms are complex communities of microorganisms adhering to a surface and grow in a four-dimensional process that resembles the development of organs. Initial colonization is followed by growth with the omission of bulk fluid and pellicle. Subsequently, differentiation occurs combined with aggregation of different species and forming mixed colonies. After continued differentiation and further growth, the microbial colonies become more structured and change in composition. Voids in the bulk form and the substratum loses some of its cells; further layers may be deposited. The final stage of biofilm development is the detachment of cells from the biofilm colony and their dispersal into the environment. This is an essential stage of the biofilm life cycle that contributes to biological dispersal, bacterial survival, and disease transmission. Like other stages of biofilm development, dispersal can be a complex process that involves numerous environmental signals, signal transduction pathways, and effectors [3].

It is estimated that about 80% of all microbial infections in humans are a direct result of biofilms. Key pathogens such as *Staphylococcus aureus*, *Pseudomonas aeruginosa*, and *Escherichia coli* have the ability to form biofilms in the human body and are associated with serious systemic diseases such as osteomyelitis, endocarditis, cystic fibrosis, pneumonia, chronic obstructive pulmonary disease, and many other systemic conditions [4,5,6,7,8,9,10,11,12]. In addition to systemic diseases, oral diseases, such as periodontitis and peri-implantitis, can also be a result of biofilm formation. For that reason, anti-biofilm agents have been introduced as treatment options. Specifically for periodontitis and peri-implantitis, commonly used anti-biofilm agents include a variety of antibiotics and the antiseptic chlorhexidine (CHX), which can be delivered systemically or locally [13,14,15,16,17]. Different systematic reviews have compared traditional non-surgical mechanical treatment for periodontitis and peri-implantitis with debridement combined with anti-biofilm agents or anti-biofilm agents alone [13,15,16,17,18]. For local delivery antibiotics, the results are still controversial [13,15,19]. Apart from antibiotics, local delivery of CHX combined with scaling and root planing (SRP) has been shown to improve the outcome of periodontal treatment [17]. Recently, antimicrobial agents isolated from animals, microbes, or plants have been introduced. An example of these agents is Chitosan, a nanotechnology product of animal origin, that has been described as biocompatible and nontoxic [20]. It has been proposed that Chitosan alone or in combination with antibiotics may be used to treat bacterial infections.

The ideal anti-biofilm approach should be able to disarm the pathogens, inhibiting an important virulence factor such as biofilm formation, without interfering with commensals viability which may cause microbial dysbiosis. Different from conventional antibiotics such as chlorhexidine, some of the novel treatment strategies for biofilm infections aim at specifically targeting unique biofilm characteristics to minimize or eliminate the drug resistance of oral biofilm. The purpose of this review is to reveal some of the novel anti-biofilm agents for the inhibition of oral biofilm, including nanoparticles, quaternary ammonium salts, and natural products.

## 2. Microbial Ecology of Dental Plaque

Saliva contains thousands of free-floating bacteria per milliliter that progressively deposit on dental/implant surfaces, first by non-specific mechanical and chemical means and then by specific interactions with surface-adsorbed saliva proteins. The initial colonizers of early dental plaque in the first few days are essentially composed of Gram-positive bacteria, mostly cocci [21]. The population then becomes increasingly complex, shifting progressively to a large Gram-negative community with the appearance of rods, filamentous organisms, vibrios, and spirochetes. Maturation of undisturbed dental plaque is very important because it is associated with the clinical development of gingival, periodontal and peri-implant mucosal inflammation [22,23]. This microbial succession is mediated by coaggregation between different bacterial species that corresponds to intergeneric specific cell-to-cell recognition via surface adhesins and receptors [24].

In more advanced disease states such as periodontitis, the diversity of the periodontal microbiota increases further. Supragingivally, it is composed of dense filament-containing plaque, while subgingivally due to a decrease in oxygen available in the surrounding environment, plaque is composed mainly of flagellated bacteria, spirochetes, and Gram-negative bacteria [25]. As previously mentioned, the early colonizers that initiate biofilm formation were mainly Gram-positive and belonged to the genera *Streptococcus* and *Actinomyces* that influence the local environment, which allows subgingival biofilm to become suitable for secondary colonizers such as *Fusobacterium nucleatum* [26,27]. This bacterium acts as a “bridging species”. Indeed, through coaggregation, it allows the adhesion of late colonizers and periopathogens like *Porphyromonas gingivalis* [24,28]. This succession during the colonization of the periodontal/peri-implant diseased crevice shows how the accumulation of commensal bacteria can induce a change in the local habitat such as the increase in pH, increase in gingival crevicular fluid (GCF), and decrease in oxygen, which allows periopathogens to colonize the periodontal/peri-implant crevice. This shift from a symbiotic microbial community to a more complex and aggressive microbiota is a risk predisposing the site to disease [29]. It is now well accepted that dental biofilms play a key role in the initiation and progression of periodontal and peri-implant diseases.

## 3. Management of Biofilm-Induced Oral Chronic Infections

The biofilm-forming capacity of bacteria is currently recognized as an important virulence determinant in the development of the various systemic infections. Bacteria have the ability to survive under various conditions of stress, including antibiotics, nutrient limitations, and immune responses [30,31]. Despite all the discoveries of the past 20 years regarding the etiopathogenesis of periodontal and peri-implant diseases, as well as the significant advances in our understanding of microbial biofilms, the incidence of these biofilm pathologies continues to rise [32,33]. Periodontitis is a biofilm-associated inflammatory disease related to a switch from a symbiotic to a dysbiotic microbiota [34]. Peri-implant diseases are associated with microbiomes that differ from those of periodontitis [35,36,37,38,39]. Control of the subgingival dysbiotic dental biofilm to restore homeostasis between the microbial community and its host remains the main purpose of currently available clinical treatments for these peri-implant and periodontal diseases. This primarily involves giving instructions for proper oral hygiene, as well as non-surgical mechanical debridement of the periodontal and peri-implant pockets [40,41]. Unfortunately, for advanced periodontal lesions with probing pocket depths of ≥7 mm, these treatments are less efficient, with about 15% showing no improvement [42].

**Figure 1:**
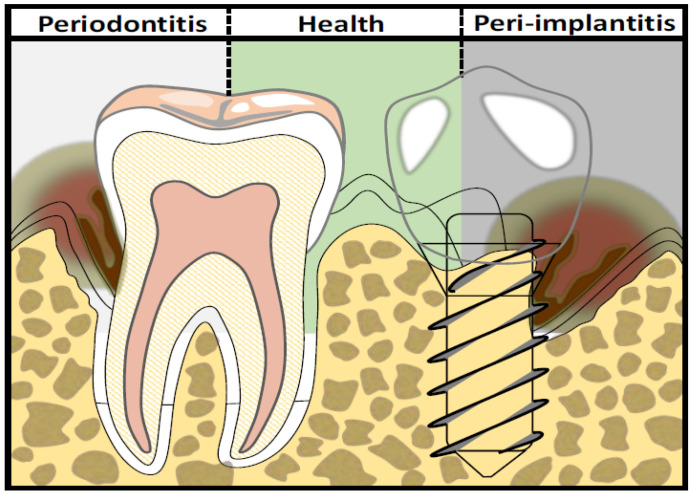
Periodontal health versus periodontitis around natural tooth structure (**left**); peri-implant health versus peri-implantitis around an implant surface (**right**).

The relative failure of mechanical treatment in peri-implantitis and periodontitis can be related to local factors, such as deep pockets, unfavorable root anatomy, or rough implant surface threads, making complete and efficient mechanical debridement difficult [43]. As illustrated in Figure 1, the comparison between periodontal health and disease around natural teeth as well as a comparison between peri-implant health and disease are primarily associated with biofilm accumulation around tooth and implant surfaces. To improve clinical results, some authors proposed the use of conventional antibiotics or local antiseptics as adjunctive treatment to the mechanical debridement of diseased pockets; their use is now recommended for the treatment of aggressive periodontitis [44].

In vitro studies have evaluated how common anti-biofilm agents affect oral biofilm. Minocycline released in agar disk cultivation has shown a reduction of *Aggregatibacter actinomycetemcomitans* [45]. Similarly, minocycline was able to inhibit the biofilm formation of *Streptococcus gordonii* and *P. gingivalis* [46]. Moreover, in an in vitro study comparing doxycycline against an amoxicillin and metronidazole regimen it was shown that doxycycline was more effective against *A. actinomycetemcomitans* [47]. Four different antibiotics (tetracycline, minocycline, doxycycline, and ofloxacin) were compared against *Prevotella intermedia* in vitro, a key bacterial component of subgingival plaque. Of these four antibiotics, ofloxacin managed to eradicate *P. intermedia* from the plaque [48]. CHX has often been used against the periodontal biofilm. CHX, at a high concentration, has been proven to be bactericidal against most oral bacteria [49]. It affects bacterial metabolism and inhibits biofilm formation [50]. However, other studies suggest that CHX alone cannot completely disrupt the periodontal biofilm and mechanical debridement is still the key component of the periodontal treatment regimen [51].

## 4. Non-Surgical Management of Periodontitis

**Figure 2:**
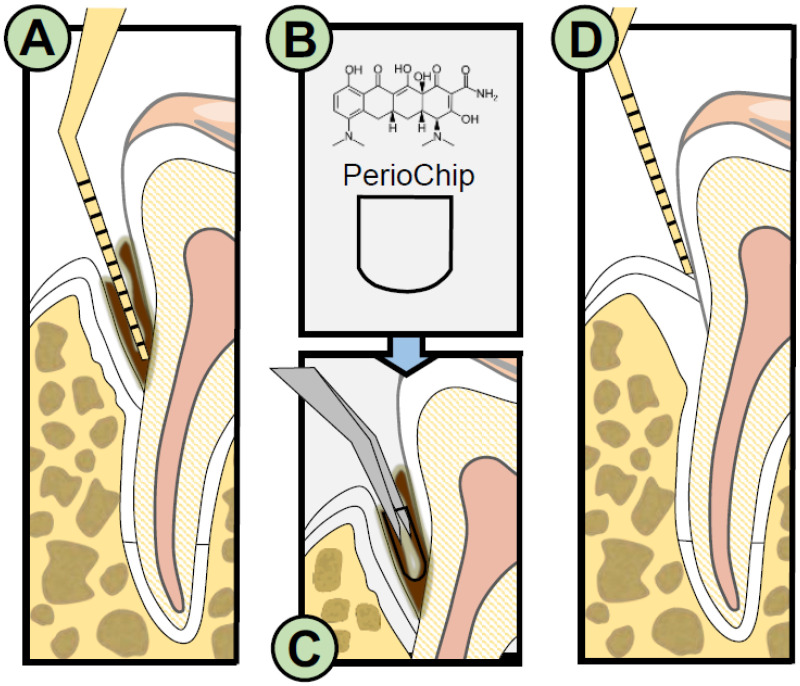
Local delivery treatment with chip containing Chlorhexidine (**A**) after non-surgical periodontal debridement to reduce biofilm; (**B**) small chip (4.5 × 3.5 mm^2^) composed of biodegradable hydrolyzed gelatin matrix, crosslinked with glutaraldehyde, also contains glycerine and water, into which 2.5 mg Chlorhexidine Gluconate is incorporated; (**C**) Perio Chip inserted into periodontal pocket; (**D**) improvement in PD and CAL with Perio Chip as an adjunct to SRP.

The traditional non-surgical treatment for periodontitis consists of SRP. Moreover, delivery of local antimicrobial agents has been proposed as an adjunct to mechanical debridement. CHX Gluconate Chip is a product that has been used in combination with SRP leading to better results compared to SRP alone [15]. Local delivery treatment with a chip containing CHX is shown in Figure 2. It has been shown that the number of pockets ≥7 mm-deep that reduced ≥2 mm was almost doubled in patients treated with SRP plus CHX Gluconate Chip compared to SRP alone [52]. Improvement of pocket depth (PD), Clinical Attachment Level (CAL) with the CHX Gluconate Chip as an adjunct to SRP has also been shown for pockets ≥ 5 mm [17]. Moreover, studies have shown mean improvement in PD between 2.3 and 3.4 mm and CAL gain 1.0–2.3 mm [53]. However, CHX Gluconate Chip does not seem to offer any additional benefit to SRP alone during the maintenance phase of periodontal treatment [54].

In addition to CHX Gluconate Chip local administration, local antibiotic delivery has been used as an adjunct to SRP. Different tetracyclines and metronidazole are the locally administered antibiotics that have been commonly used, resulting in significant improvement in PD and CAL [19,55]. Local antibiotic delivery reduces PD by 1.5 mm on average, while other studies have shown that tetracycline, doxycycline, minocycline, and CHX offer a reduction of 0.7 mm, 0.5 mm, 0.4 mm, and <0.4 mm, respectively [16,19]. Minocycline is delivered in the form of spheres or gel, is bioresorbable, and its additional benefit in PD reduction is 0.9 mm in pockets ≥ 7 mm [13,56]. Tetracycline is administered as fibers and they have to be removed after 7–10 days. The additional benefit of administered tetracycline in combination with SRP compared to SRP alone is still controversial, but it has been reported that administration in pockets with PD ≥ 5 mm improves significantly PD and CAL measurements along with other clinical parameters [13,56,57]. Doxycycline applied locally as a gel has been shown to improve PD and CAL, as well as control clinical inflammation in treated patients [58]. Metronidazole local delivery when used as an adjunct to SRP did not show any additional improvements to SRP [13,56]. Generally, local delivery antibiotics are recommended in pockets ≥ 5 mm especially in patients that do not respond as expected to traditional SRP [14,59]. The molecules used in local delivery as adjuncts in treatment of periodontitis are listed in Table 1.

## 5. Clinical Signs and Management of Peri-Implantitis

Peri-implantitis is an inflammatory disease characterized by biofilm deposition on the implant surfaces. Bleeding on probing (BoP) with or without suppuration, visual signs of inflammation, increased PD, and increased radiographic bone loss from the initial bone remodeling are the characteristic features of peri-implantitis [60,61]. Smoking and history of periodontitis are considered important risk indicators for peri-implantitis [62].

Regarding peri-implantitis, surgical and non-surgical treatment options have been proposed. As an adjunct to those treatments, local antimicrobial agents are frequently used. Biofilm removal with plastic instruments followed by tetracycline HCl fiber application for 10 days showed significant PD reduction from 6 to 4.1 mm without significantly affecting the intrabony component of the peri-implant lesion after one year [63]. Moreover, *P. intermedia/nigrescens*, *Fusobacterium* sp., *Bacteroides forsythus*, and *Campylobacter rectus* were significantly reduced. Similarly, a randomized control study has shown that local application of Minocycline with peri-implantitis surgery accompanied by application at 1, 3, and 6 months after surgery resulted in 66.7% PD reduction to <5 mm compared to 36.3% reduction in the control group [64]. Local administration of minocycline spheres may also significantly reduce the levels of *A. actinomycetemcomitans* in peri-implantitis cases and maintain these low levels of *A. actinomycetemcomitans* for up to one year. On the other hand, reductions of *Tannerella forsythia, P. gingivalis*, and *Treponema denticola* treated with minocycline spheres could only be maintained for 180 days.

Multiple reports from reviews also support the use of local antimicrobial agents. The use of CHX or minocycline spheres can improve both PD and BoP as well as reduce microbial pathogens [40,43,65]. However, when CHX gel was compared with minocycline spheres, it was shown that minocycline spheres therapy managed to improve both PDs and BoP scores when compared to CHX gel. CHX gel only managed to reduce BoP [43,65,66,67]. The combination of CHX rinse with local application of a slow-release doxycycline gel was found to improve the clinical parameters around implants with peri-implantitis [62,67]. On the other hand, a randomized control clinical trial suggested that local administration of CHX for implant surface disinfection during peri-implantitis surgery had no significant additional clinical benefit on PDs and BoP [68].

Subgingival irrigation as a nonsurgical treatment of periodontal and peri-implant diseases remains controversial [69]. There is a lack of randomized controlled clinical trials, and the data do not allow for distinguishing between the relative efficacy of various available treatment methods [70]. Therefore, the development of new strategies to better treat severe periodontitis and peri-implantitis are still needed. To this end, one approach would be to investigate new clinical strategies to control more efficiently the periodontal and peri-implant biofilms and pathogens associated with periodontitis and peri-implantitis. Molecules used in local delivery as adjunctive treatment of peri-implantitis are listed in Table 2.

## 6. Emerging Anti-Biofilm Strategies

Periodontal disease is primarily of bacterial etiology, from multispecies biofilms of Gram-negative anaerobic microorganisms. The deleterious effects are caused by the resultant inflammatory response to the microbial insult. Therefore, the development of a treatment that combines anti-biofilm activity with anti-inflammatory activity would be of great utility. Naturally occurring antimicrobial peptides (AMPs) such as defensins have been suggested as a novel alternative to standard antibiotics because they exhibit broad-spectrum activity as well as a variety of immunomodulatory activities with little development of antibiotic resistance. However, AMP development as exogenous antibiotics is difficult for large-scale production due to limited availability. Moreover, AMPs have poor tissue distribution and potential systemic toxicity. However, the development of synthetic AMP molecule mimetics has provided an unlimited supply of such agents. An example of small molecule AMP mimetics is mPE, which mimics natural AMPs and has shown promising results with potent activity against key periodontal pathogens such as *P. gingivalis* and *A. actinomycetemcomitans*. Importantly, this compound can also inhibit the lipopolysaccharide (LPS)-mediated induction of TNF-α from macrophages and, thus, mitigate the inflammatory response of bacterial biofilm [71].

The emergence of more natural anti-plaque agents that are safer for humans and more specific against oral pathogens would be more desirable. It has been shown that a compound named Macelignan, one of the bioactive compounds found in nutmeg, has a potent anti-biofilm activity against oral primary colonizers such as *Streptococcus mutans, Streptococcus sanguis*, and *Actinomyces viscosus*. While this study was in vitro, the promising role of this compound as an anti-biofilm agent for treating periodontitis and peri-implantitis should be substantiated further through clinical studies [72,73]. The ideal anti-biofilm approach is to promote the dispersion of formed biofilms, eliminate pathogens, and impede the formation of new biofilms while avoiding the elimination of commensals, which may cause microecology dysbiosis [74,75,76]. Some of the novel treatment strategies for biofilm infections target unique biofilm characteristics to minimize or eliminate the drug resistance of oral biofilm [77].

## 7. Synthetic Agents

**Figure 3:**
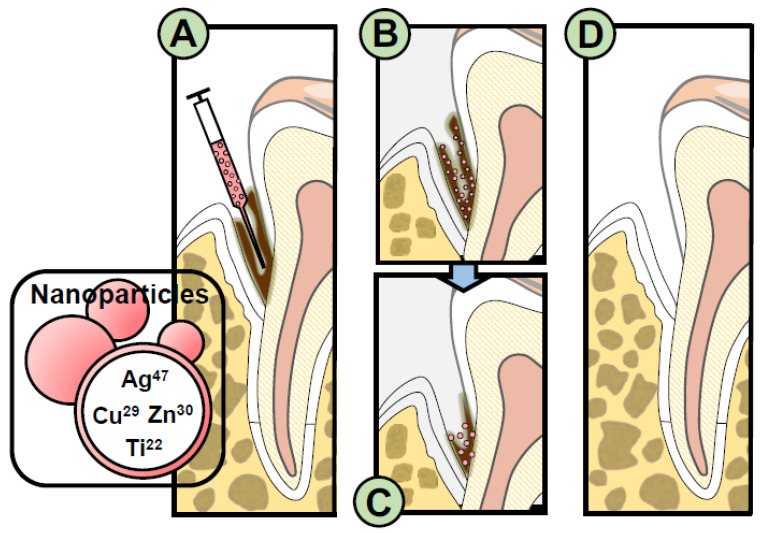
Illustration for the treatment of periodontitis with nanoparticles containing anti-biofilm molecules (**A**) Delivery of anti-biofilm nanoparticles into diseased sites; (**B**) ability of nanoparticles to attach to and incorporate into biofilm and release active agents such as CHX for up to 48 h; (**C**) gradual reduction of multispecies biofilm; (**D**) elimination of biofilm and established state of health. A similar therapeutic approach could be used for the treatment of peri-implantitis.

Another anti-biofilm approach would be the use of nanoparticles. Nanoparticles such as silver, copper oxide, zinc oxide, titanium oxide, and graphene, could be used to control biofilm formation [78,79,80]. Additionally, quaternary ammonium polyethyleneimine, chitosan, and silica nanoparticles have also been suggested effective in controlling biofilms [78,81,82]. Silver nanocoating applied directly on dentin, tooth, or implant surfaces can prevent biofilm formation on dentin and inhibit bacterial growth. This suggests that Silver Nanoparticles (AgNPs) could protect the tooth from pathogenic dental plaque and secondary caries when applied as a dentin coating [83]. Importantly, AgNPs exhibit the anti-biofilm potential against *Enterococcus faecalis*, which is known as one of the main causes of secondary and persistent endodontic infections. An illustration for the treatment of periodontitis with nanoparticles containing anti-biofilm molecules is shown in Figure 3.

Chitosan is a nontoxic natural cationic polysaccharide with characteristics of adhesiveness, antimicrobial activity, biocompatibility, and biodegradability [84,85]. The bactericidal property of chitosan nanoparticles (CNPs) is attributed to their high surface charge density, which permits them to interact with the negative charge surface of bacterial cells, causing bacterial cell death [86]. Ag-conjugated CNPs can inhibit the growth and adherence of *P. gingivalis* and reduce the biofilm formation on dental implants, thus, representing a potential anti-biofilm coating material for titanium dental implants [87].

Other promising nanoparticles are the Mesoporous Silica Nanoparticles [MSNs). When encapsulated with the antimicrobial agent CHX they can attach on microbes and release CHX for up to 48 h [88]. Additionally, MSNs have demonstrated potent antibacterial activity against *S. mutans*, *Streptococcus sobrinus*, *F. nucleatum*, *A. actinomycetemcomitans* either in a planktonic state or in monospecies biofilms. MSNs have been shown to suppress multispecies biofilms of *S. mutans*, *F. nucleatum*, *A. actinomycetemcomitans*, and *P. gingivalis* for up to 72 h [89].

## 8. Natural Products

Natural products have been shown to exhibit biological activities that make them promising candidates as alternative or adjunctive therapies in reducing dysbiotic oral biofilm [90]. Tea has been proven to have many health benefits including antioxidant, antidiabetic, hypocholesterolemic, antibacterial, anti-inflammatory, and cancer-preventive properties [91,92,93]. Its activity against oral biofilm formation is mainly attributed to polyphenols [91,94,95,96,97]. An epidemiologic study demonstrated that frequent consumption of green tea was positively correlated with good periodontal health [98]. Consistently, in vitro studies demonstrated polyphenol compounds present in green tea could significantly inhibit the growth, adherence, and biofilm formation of *P. gingivalis*, as well as suppress the activity of collagenase and matrix metalloproteinases [99,100,101,102]. In addition, phenol compounds enhance gingival keratinocyte integrity to protect from invasion or adherence of *P. gingivalis* [102]. Cranberry, a highly nutritious fruit, is known for its high concentration of total polyphenols, making it an excellent antioxidant and has been reported to be beneficial for fighting bacterial infection [103]. Cranberries have demonstrated potential inhibitory effect against bacteria related to dental caries and periodontal diseases [104]. Proanthocyanins (PACs) and flavonols are the most active components of the cranberry that can disrupt biofilm formation of *S. mutans* [105,106,107]. PACs are also effective in the prevention and management of periodontitis. They reduce biofilm formation, bacterial adherence, and invasiveness to the human epithelial cells and proteinase activity of *P. gingivalis* [108,109].

## 9. Conclusions

The present review has confirmed the extensive previous and current research efforts related to the use of local antimicrobial agents in periodontal and peri-implant therapy. However, many challenges and opportunities lie ahead. These include the use of nanomaterials, such as silver nanoparticles, instead of conventional antimicrobial/anti-biofilm materials, and the combination of one or more ions to take advantage of the synergistic effects of multiple ions. In addition to synthetic agents, natural products such as polyphenol compounds present in green tea and cranberry fruit have promising anti-biofilm properties. Nevertheless, further research is needed to identify and elucidate in depth the biocidal mechanisms by which different ions act against the specific pathogens, and to provide evidence and quantitative experimental data to identify these ions or nanoparticles. The anti-biofilm molecules, ions, or nanoparticles could then potentially serve more effectively as anti-biofilm agents in the control of periodontal and peri-implant diseases, either in suspensions or after incorporation into biomedical devices (biomaterials).

## Figures and Tables

**Table 1:** Molecules used in local delivery as adjunctive treatment of periodontitis.

Locally Administered Antimicrobial	Active Agent	Results	References
Periochip	Chlorhexidine gluconate 2.5 mg	In combination with SRP reduced PDs > 2 mm compared to SRP alone after 9 months	[52]
Periochip	Chlorhexidine gluconate 2.5 mg	In combination with SRP improved the results of periodontal treatment compared to SRP alone	[17]
Periochip	Chlorhexidine gluconate 2.5 mg	In combination with SRP reduced PDs and resulted in CAL gain compared to SRP alone after 9 months	[15]
Periochip	Chlorhexidine gluconate 2.5 mg	Chip alone showed no statistically significant differences compared to SRP during maintenance period	[54]
Periochip	Chlorhexidine gluconate 2.5 mg	In combination with SRP studies showed statistically significant improvements to PD and CAL gain compared to SRP alone	[19]
Periochip, PerioCol	Chlorhexidine gluconate 2.5 mg	In combination with SRP most studies showed non statistically significant improvements compared to SRP alone	[53]
PerioChip	Chlorhexidine gluconate 2.5mg	In combination with SRP studies showed statistically significant improvements to PD and CAL gain compared to SRP alone	[14]
Periochip	Chlorhexidine chip	In combination with SRP studies showed statistically significant improvements to PD and CAL gain compared to SRP alone	[16]
Chlosite	Chlorhexidine gluconate gel 0.5% or 1%	In combination with SRP studies showed no statistically significant improvements to PD and CAL gain compared to SRP alone	[14]
Chlosite	Chlorhexidine gluconate 0.5% or 1%	In combination with SRP most studies showed statistically significant improvements to PD and CAL gain compared to SRP alone	[53]
Atridox	Doxycycline 10% or 50 mg	Atridox alone showed equivalent results with SRP after 9 months	[15]
Atridox	Doxycycline 10% or 50 mg	In combination with SRP results related to PDs and CAL compared to SRP alone were inconclusive	[53]
Atridox	Doxycycline gel 8.8%	In combination with SRP studies showed statistically significant improvements to PD and CAL gain compared to SRP alone	[14]
-	Tetracycline and citric acid gel	In combination with SRP statistically significant improvements of PD compared to SRP alone	[19]
Periodontal Plus AB	Tetracycline fibers 8%	In combination with SRP most studies showed statistically significant improvements to PD and CAL gain compared to SRP alone	[53]
-	tetracycline-loaded ethylene vinyl acetatefibers [TNC]	In combination with SRP studies showed statistically significant improvements to PD compared to SRP alone	[56]
-	Tetracycline	In combination with SRP studies showed statistically significant improvements to PD and CAL gain compared to SRP alone	[57]
Arestin	Minocycline 1 mg	In combination with SRP reduced PDs compared to SRP alone after 9 months	[15]
Arestin	Minocycline 1 mg	In combination with SRP most studies showed statistically significant improvements to PD and CAL gain compared to SRP alone	[53]
Dentomycin	Minocycline gel 2%	In combination with SRP studies showed statistically significant improvements to PD and CAL gain compared to SRP alone	[14]
Elyzol	Metronidazole gel 25%	In combination with SRP results related to PDs and CAL compared to SRP were inconclusive	[53]
-	Metronidazole gel 25%	In combination with SRP studies showed no statistically significant improvements to PD compared to SRP alone	[56]
Elyzol	Metronidazole gel 25%	In combination with SRP studies showed statistically significant improvements to PD compared to SRP alone	[14]

Abbreviations: PD = Pocket depth; CAL = Clinical Attachment level; SRP = Scaling and Root Planing.

**Table 2:** Molecules used in local delivery for the treatment of peri-implantitis.

Locally Administered Antimicrobial	Active Agent	Results	References
Corsodyl	Chlorhexidine gel 1%	SRP with minocycline reduced statistically significantly the PDs at 12 months Compared to SRP with CHX.	[65]
-	Chlorhexidine gel	Clinical parameters were statistically significantly improved.	[43]
-	Chlorhexidine gel 1%	SRP with minocycline reduced statistically significantly the PDs at 90 days. Compared to SRP with CHX.	[66]
-	Chlorhexidine solution 0.2%	The use of CHX to disinfect the implant surface during surgery had no additional benefit.	[68]
Actisite	Tetracycline HCl-containning fibers	PDs were statistically significantly reduced at 12 months.	[63]
-	Tetracycline fibers	Clinical parameters were statistically significantly improved.	[43]
Periocline	2% minocycline HCl gel	Surgery with minocycline statistically significantly reduced PDs and increased supporting bone levels compared to surgery alone.	[64]
-	1mg minocycline HCl	SRP with minocycline reduced statistically significantly the PDs at 90 days. Compared to SRP with CHX.	[66]
Arestin	Minocycline 1 mg	SRP with minocycline reduced statistically significantly the PDs at 12 months Compared to SRP with CHX.	[65]

Abbreviations: PD = Pocket Depth; CHX = Chlorhexidine. SRP = Scaling and Root Planing.

## Data Availability

Not applicable.

## Sample Availability

Not available.
